# Anæsthesia

**Published:** 1849-06

**Authors:** 


*Anæsthesia.* From Dr. Griscom's Report on Obstetrics, to the Phila. Coll. of Physicians.

We now reach the subject which, for the period our report refers to, has of all others claimed the most interest; we allude to the exhibition of ether and chloroform by inhalation, for the purpose of producing anæsthesia during parturition. On the 19th of the first month, (January,) 1847, Professor Simpson, of Edinburg, administered ether to a woman in labor, for the purpose of rendering her insensible to pain; it being a case of deformed pelvis, in which the doctor intended to extract the child by turning; he regarded it of little importance whether etherization checked the uterine contractions or not; the result shewed that while consciousness to pain was destroyed, the muscular contractions of the uterus continued. This case was reported to the Obstetric Society of that place the next day. In the succeeding three weeks, Dr. Simpson exhibited ether in "several cases of natural labor, and in one forceps case," and drew from them the following conclusions, which he laid before the same Society:

1. That the inhalation of ether procured for the patient a more or less perfect immunity from the conscious pain and suffering attendant upon labor.

2. That it did not however, diminish the strength or regularity of the contractions of the uterus.

3. That, on the other hand, it apparently (more especially when combined with ergot) sometimes increased them in severity and number.

4. That the contraction of the uterus after delivery seemed perfect and healthy when it was administered.

5. That the reflex assistant contractions of the abdominal muscles, &c., were apparently more easily called into action by artificial irritation and pressure on the vagina, &c., when the patient was in an etherized state.

6. That its employment might not only save the mother from more pain in the last stages of labor, but might probably save her also, in some degree, from the occurrence and consequences of the nervous shock attendant upon delivery, and thereby reduce the danger and fatality of childbed; and

7. Its exhibition did not seem injurions to the child.

A few weeks subsequently he reported further, that the fœtal heart was but little, if at all, influenced by full and prolonged etherization, either during pregnancy or labor; and that the mother during labor might be kept etherized, if required, for one, two, three, or more hours; he having given it in one case about six, and in another, four hours before delivery.—Under very deep etherization, the uterine contractions were somewhat modified, but recurred immediately in full force as the effect passed off. Dr. Simpson had hitherto seen no traceable injury to mother or child. Immediately after the publication of these results by Prof. Simpson, the subject was investigated by various observers throughout Great Britain and on the continent; amongst the most distinguished of these were Dr. Murphy, Professor of Midwifery in the University College, London, Dr. Prothero Smith, Baron Dubois of Paris, Professor Villeneuve of Marseilles, Stoltz, of Strasburg, Martin, of Jena, Siebold, of Gottingen, and Grenser, of Leipsic.

In a paper read before the Harverian Society, London, February 5th, 1848, Professor Murphy gives the details of seven cases in which he exhibits chloroform, and draws from them the following conclusions:

1. It does not interfere with the action of the uterus, unless it be given in very large doses, which is never necessary.

2. It causes a greater relaxation in the passages and perineum; the mucous secretion from the vagina is also increased.

3. It subdues the nervous irritation caused by severe pain, and restores nervous energy.

4. It secures the patient perfect repose for some hours after her delivery.

These three last effects consequently render an operation much easier to perform, and the recovery of the patient afterwards much more probable.

5. The order of its effects on the vital functions seems to be — loss of sensation, partial loss of voluntary motion, loss of consciousness, complete loss of the voluntary motion, stertorous respiration, loss of involuntary motion, cessation of the action of the uterus, of respiration, of the action of the heart.

6. Its injurious effect, when an ordinary dose is given, seem to depend on constitutional peculiarities, or on improper management. Much excitement about the patient may render her violent; catalepsy has occurred in some, clonic contractions in others. Some patients are slow in recovering from the effects of a large dose; they remain giddy during the day, and sometimes faint when they stand upright.

Professor Dubois's conclusions as officially reported are, that

1. The inhalations of ether can annul the pain of obstetrical operations.
2. It can suspend the physiological pains of labor.
3. It destroys neither the uterine contraction nor the contraction of the abdomical muscles.
4. It diminishes the natural resistance of the perineum.
5. It does not appear to act unfavorably on the health or life of the infant.

In America, Dr. Channing, of Boston, has bestowed much labor and care on the investigation of this subject. An octavo volume of four hundred pages, published the latter part of the past year, is the result of his assiduity. This work presents the subject to the profession in a manner calculated to claim attention. The cases referred to by Dr. Channing, in which ether or chloroform were used, amount to five hundred and sevent-four. They occurred in his own practice, and in that of upwards of forty other respectable practitioners, many of them of high standing.

Dr. C. says, "in five hundred and sixteen cases of labor, embracing among them all the circumstances belonging to this process, except instrumental and manual aid; in all these cases of labor, accomplished during etherization, we have not a case in which the mother did not do well; cases of still birth are referred to, but these, as we have seen, had no connection with etherization." "My confidence in the remedy of pain in this class of labor, (natural) increases daily. The confidence the public have in its invaluable agency also increases; and, reasoning from direct observation of its effects, is there any place for doubt of its ultimate and universal employment."—Dr. C. further says, "very little is offered in this volume of the comparative statistics of midwifery practice, with and without etherization, and it seemed hardly necessary to do this, for I have not reported a case, (and I have reported all which I have been able to obtain,) of untoward result to either mother or child, which by any just reasoning can be ascribed to etherization."

Other observers in various parts of our country, have confirmed these views and reults.

Amongst the numerous correspcndents, whose authority is quoted by Dr. Channing, we find that of Prosessor John Ware, of Boston, who has given ether in two cases of obstetrics, and is favorable to the inhalation of both ether and chloroform: of Professor Bigelow, of Boston, who had exhibited ether in eight cases, and chloroform two; and is favorable to both: Dr.

Pierson, of Salem, Mass., who has given ether in three cases; opinion favorable: of Dr. Putnam, of Boston, who reports seventeen cases of ether, seven of chloroform; opinion favorable: of Dr. Storer, who reports thirty-eight cases of ether, two of chloroform; opinion favorable: of Dr. Bartlett, of New Bedford; has used ether in two cases; is favorable to its use in certain cases, but not in all: of Dr. Jarvis of Dorchester; has used ether in five cases; opinion favorable: of Professor Roby, of Baltimore; has used chloroform in one case; opinion favorable: of Dr. A. K. Goodwin, of New York; has used chloroform in three cases; opinion favorable: and of Dr. Bartlett, of Concord, Mass., who has used ether in three cases; but gives no decision.

Dr. Burwell, of Buffalo, gives, *in the Buffalo Med. Jour.*, a statement of fifteen cases of midwifery in which chloroform was administered; he divides them into two classes: 1st, those in which anæsthesia was complete, or nearly so. 2d, those in which complete consciousness was retained of surrounding circumstances, or only momentarily lost. Eight cases came under the first division. The second class of cases demonstrate, in Dr. Burwell's opinion, the possibility of so exhibiting chloroform that consciousness is retained for surrounding circumstances, and yet the suffering so diminished as to be of little importance; this effect has been noticed by Dr. Channing and others, and was fully exemplified in one of the cases in which ether was exhibited by your reporter.

Profestor Lindsley, of Washington, D. C., says, "that sufficient evidence has now been advanced in favor of etherization in midwifery practice, it having been employed in probably two thousand cases, without a single fatal result, to render it the duty of the profession to give it further trial; to experiment with it cautiously and judiciously, in order to see if we cannot finally arrive at general laws and principles which will enable us to administer it without danger or apprehension." Dr. Clark, of this county, has published, in the Medical Examiner, several cases in which ether was exhibited with favorable results, and which have already been reported to this College.

Besides these, we are not aware that any cases have been published by residents of this city and its vicinity. But we understand from our friends, that several of them have given the practice a partial trial, without any untoward effects having resulted. Your reporter has used it in but two cases, one a forceps case, and the other of natural labor. In both, strong expressions of satisfaction and gratitude were used by the patients at the time, and afterward, and their recovery was rapid and uninterrupted; in the forceps case the increased secretion of vaginal mucus after the induction of anæsthesia, was very striking.

Nearly two years have now elapsed since the practice was introduced by Professor Simpson; it has been tested by careful and competent observers in Europe and America; and most, if not all, who have published the results of their observations on this subject, agree that the suffering of labor can be greatly ameliorated, or entirely abolished, by the inhalation of either sulphuric ether or chloroform; that the expulsive efforts of the uterus are not interfered with to any considerable extent: that the general condition of the system for safe and rapid delivery is improved; that the exhaustion which usually follows labor is diminished; and that no serious disadvantage has hitherto resulted to mother or child. On the other hand, it has been clearly shown by the experiments of Wakely and others, that both ether and chloroform, if improperly or excessively exhibited, are highly dangerous, and hence it becomes a question whether the immunity from danger which has hitherto attended the exhibition of these agents in midwifery is the result of an adherence to certain fixed principles, or is accidental? This, it appears to your reporter, is the only question of importance in regard to it, which remains to be settled, and he has no hesitation in asserting, that if extended experience settles it in favor of the safety of the use of either agent, an inestimable boon will have accrued to mankind; not that it would be proper, even then, to resort to it in all cases, but if the practitioner can *feel himself at liberty to resort* to it without apprehension of danger should the pangs of childbirth become intolerable, a vast amount of mental disquietude, as well as physical agony will be saved to those females who place themselves under his care.

